# Selective amyloid-β lowering agents

**DOI:** 10.1186/1471-2202-9-S2-S4

**Published:** 2008-12-03

**Authors:** Michael S Wolfe

**Affiliations:** 1 Center for Neurologic Diseases, Brigham & Women's Hospital and Harvard Medical School, Boston, MA 02115, USA

## Abstract

The amyloid-β peptide (Aβ), implicated in the pathogenesis of Alzheimer's disease (AD), is produced through sequential proteolysis of the Aβ precursor protein (APP) by β- and γ-secretases. Thus, blocking either of these two proteases, directly or indirectly, is potentially worthwhile toward developing AD therapeutics. β-Secretase is a membrane-tethered pepsin-like aspartyl protease suitable for structure-based design, whereas γ-secretase is an unusual, heterotetrameric membrane-embedded aspartyl protease. While γ-secretase inhibitors entered clinical trials first due to their superior pharmacological properties (for example, brain penetration) over β-secretase inhibitors, it has since become clear that γ-secretase inhibitors can cause mechanism-based toxicities owing to interference with the proteolysis of another γ-secretase substrate, the Notch receptor. Strategies for targeting Aβ production at the γ-secretase level without blocking Notch signalling will be discussed. Other strategies utilizing cell-based screening have led to the identification of novel Aβ lowering agents that likewise leave Notch proteolysis intact. The mechanism by which these agents lower Aβ is unknown, but these compounds may ultimately reveal new targets for AD therapeutics.

## 

The formation of the amyloid-β peptide (Aβ) from the Aβ precursor protein (APP) is a critical molecular event in the pathogenesis of Alzheimer's disease (AD). For this reason, the proteases that produce Aβ from this integral membrane protein are considered key targets in the prevention and treatment of AD [1]. β-Secretase generates the amino terminus of Aβ, shedding the large ectodomain (β-APP_s_) and leaving a 99 residue carboxy-terminal fragment (C99) in the membrane. C99 is cleaved in the middle of its transmembrane domain by γ-secretase to produce Aβ. γ-Secretase produces carboxy-terminal variants of Aβ, primarily a 40-residue peptide (Aβ40), but also a small proportion of a 42 residue variant (Aβ42), as well as other minor species. Aβ42 is much more prone to aggregation than Aβ40, and Aβ42 is the major Aβ species found in cerebral plaques that characterize the AD brain.

β-Secretase is a membrane-tethered enzyme in the pepsin family of aspartyl proteases and primarily expressed in the brain [2]. Knockout of this enzyme in mice is not lethal and prevents Aβ production in the brain [3,4]. Nevertheless, concern about β-secretase as a target has been raised by the finding that the myelin sheath of peripheral nerves of these knockout mice are much thinner due to the important role β-secretase plays in cleaving neuregulin-1 [5]. The extracellular catalytic domain of β-secretase has been successfully crystallized with bound inhibitors, enabling structure-based design [6]. However, the long, shallow active site of β-secretase has proved challenging in developing inhibitors with appropriate pharmacological properties, in particular the ability to cross the blood-brain barrier. Potent inhibitors tend to be too large and peptide-like, although this problem is gradually being overcome [7], and the first β-secretase inhibitors are poised to enter clinical trials.

In contrast to β-secretase, the ubiquitously expressed γ-secretase is a complex of four different integral membrane proteins essential to the protease: presenilin (PS), Nicastrin, Aph-1, and Pen-2 [8]. These proteins assemble, resulting in the cleavage of PS into an amino-terminal fragment and a carboxy-terminal fragment, a necessary step in the maturation of wild-type PS into an active component of γ-secretase. PS contains two completely conserved transmembrane aspartates that are essential for γ-secretase activity and part of the compelling evidence suggesting that PS is a novel, membrane-embedded aspartyl protease. Although PS is apparently the catalytic component of γ-secretase, it nevertheless requires the other three components to become an active protease and maintain activity.

Unlike the situation with β-secretase, identification of highly potent inhibitors of γ-secretase that readily penetrate biological membranes has not been especially problematic. However, these compounds also interfere with the processing of other substrates of this protease besides APP [9], raising serious concerns about selectivity and toxicity. γ-Secretase can cleave a number of different single-pass membrane proteins, including Erb-B4, E- and N-cadherins, CD44, the low density lipoprotein receptor, Nectin-1, and the Notch receptor ligands Delta and Jagged. However, the most pharmacologically relevant alternative substrate is the Notch receptor itself. Signalling from this receptor plays a role in a variety of cell differentiation events from embryogenesis into late adulthood. The Notch signal is initiated by interaction with a cognate ligand that induces shedding of the extracellular portion of the receptor. The remaining membrane-bound stub is then processed by PS/γ-secretase to release an intracellular domain that translocates to the nucleus and directly interacts with certain transcription factors, thereby regulating gene expression. Because γ-secretase is essential for Notch signaling, inhibitors of this protease can interfere with cell differentiation. Indeed, treatment of mice with γ-secretase inhibitors over time can cause severe gastrointestinal toxicity and compromise the proper maturation of B- and T-lymphocytes [10,11]. Thus, the ability to selectively block APP proteolysis by γ-secretase without affecting the proteolysis of Notch is a major goal toward realizing practical therapeutics for AD.

Two types of compounds appear to selectively modulate γ-secretase activity via direct interaction with the protease or its substrate. The first of these are certain non-steroidal anti-inflammatory drugs (NSAIDs), reported in 2001 by the laboratories of Todd Golde and Edward Koo to be capable of skewing the production of Aβ, lowering the production of Aβ42, and increasing the formation of a shorter 38 residue variant (Aβ38) [12]. These compounds include ibuprofen, indomethacin, and sulindac sulfide. The effects of these compounds were demonstrated in isolated membranes [13], suggesting that the compounds work directly (that is, on enzyme or substrate) instead of through an indirect effect on a signaling or metabolic pathway [14]. Evidence from our laboratory shows that these compounds can also shift the site of substrate proteolysis catalyzed by signal peptide peptidase [15]. Because signal peptide peptidase is a PS homolog that does not require additional protein cofactors, the implication is that the NSAID binding site is on PS. On the other hand, evidence from the Golde laboratory supports the APP substrate itself, specifically its juxtamembrane region, as the direct binding site, which would explain the selectivity of these compounds for APP versus Notch [16]. Understanding how this subset of NSAIDs selectively affects APP processing by γ-secretase is important, as one of these compounds, R-flurbiprofen (tarenflurbil; Figure 1), recently failed in late-stage clinical trials for the treatment of AD due to lack of efficacy. A clearer mechanistic understanding may suggest structural changes to improve the potency of this class of compounds.

**Figure 1:**
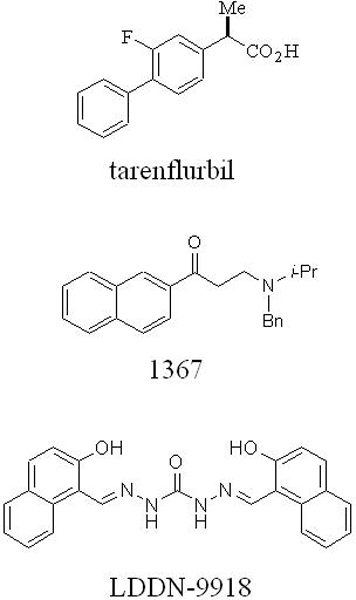
Selective amyloid-β lowering agents that do not affect Notch processing. Tarenflurbil and 1367 work in purified γ-secretase assays, whereas LDDN-9918 was identified from a cell-based screen and works through an unknown mechanism.

Certain kinase inhibitors can also selectively affect Aβ production at the γ-secretase level with little or no effect on Notch proteolysis. Because ATP was found to augment the γ-secretase cleavage of C99 to Aβ, the Greengard laboratory at Rockefeller University tested kinase inhibitors for their ability to prevent Aβ production. The Abl kinase inhibitor imatinib (Gleevec™) was found to block Aβ formation without affecting Notch [17]. This action of imatinib was not due to an interaction with Abl kinase, although it was assumed to be blocking some membrane-associated kinase. Subsequently, our laboratory found that an extract from the drug's capsules (but not imatinib itself) could inhibit Aβ production from purified γ-secretase while leaving the proteolysis of Notch unaffected [18]. An inhibitor of Janus kinase 3 (Jak3) was also found to show selective inhibition on purified γ-secretase (compound 1367; Figure 1). Further experiments revealed a nucleotide binding site on the γ-secretase complex. For example, affinity-labeling with a photo-reactive azido-substituted ATP led to its covalent attachment to the PS1 carboxy-terminal fragment. This labeling was prevented by the imatinib extract and the Jak3 inhibitor, but not by a transition-state analogue inhibitor (that is, directed to the active site). These findings suggest a specific competition with ATP for binding to the γ-secretase complex at an allosteric site.

In parallel with the above efforts, we have established a cell-based screen to identify Aβ lowering agents that work by novel mechanisms (as opposed to direct inhibition of β- or γ-secretase). Specifically, we stably co-expressed APP with a heterologous transcription factor fused to its carboxyl terminus along with a luciferase reporter, the expression of which would be turned on when APP underwent proteolytic processing and released its cytosolic domain [19]. Screening of some 65,000 drug-like compounds using this cell-based assay led to the identification of compounds that were found to lower Aβ production in secondary assays, and to leave Notch proteolysis unaffected in tertiary assays. One of these compounds is the symmetrical naphthylene-containing bis(carbohydrazone), LDDN-9918 (Figure 1). Structure-activity studies have led to the development of a smaller, more soluble, and more drug-like derivative (S Choi, P Maiti, T Gainer, M Glicksman, M Wolfe, and G Cuny, unpublished results). Elucidation of the mechanism by which this compound lowers Aβ may lead to the identification of new druggable targets for AD.

## List of abbreviations used

Aβ: amyloid-β; AD: Alzheimer's disease; APP: Aβ precursor protein; NSAID: non-steroidal anti-inflammatory drug; PS: presenilin.

## Competing interests

The author declares that they have no competing interests.

## Authors' contributions

This review article was written entirely by MSW.
